# Evaluation and validation of the diagnostic value of the apparent diffusion coefficient for differentiating early-stage endometrial carcinomas from benign mimickers at 3T MRI

**DOI:** 10.18632/oncotarget.18553

**Published:** 2017-06-16

**Authors:** Xue Wang, Yu Zhao, Yumin Hu, Yongjin Zhou, Xinjian Ye, Kun Liu, Guanghui Bai, Anna Guo, Meimei Du, Lezhen Jiang, Jinhong Wang, Zhihan Yan

**Affiliations:** ^1^ Department of Radiology, The Second Affiliated Hospital and Yuying Children's Hospital of Wenzhou Medical University, Wenzhou 325027, China; ^2^ Department of Gynecology and Obstetrics, The Second Affiliated Hospital and Yuying Children's Hospital of Wenzhou Medical University, Wenzhou 325027, China; ^3^ Department of Medical Imaging, Shanghai Mental Health Center, Shanghai Jiao Tong University School of Medicine, Shanghai 200030, China

**Keywords:** magnetic resonance imaging, endometrial lesions, diffusion-weighted imaging, apparent diffusion coefficient, diagnostic accuracy

## Abstract

Previous researchers obtained various apparent diffusion coefficient (ADC) cutoff values to differentiate endometrial carcinoma from benign mimickers with 1.5T magnetic resonance imaging (MRI). Few studies have used 3T MRI or validated the effectiveness of these cutoff ADC values prospectively. This study was designed in two stages to obtain a cutoff ADC value at 3T MRI and to validate prospectively the role of the ADC value. First, we conducted a retrospective study of 60 patients to evaluate the diagnostic value of ADC by obtain a theoretical cutoff ADC value for differentiating between benign and malignant endometrial lesions. Student's t test revealed that ADC values for stage I endometrial carcinomas were significantly lower than those for benign lesions. The area under the curve value of the receiver operating characteristic curve was 0.993, and the cutoff ADC value was 0.98 × 10^−3^ mm^2^/s. The sensitivity, specificity, and overall accuracy of diagnosing stage I endometrial carcinoma were 100%, 97.1%, and 98.3%, respectively. Second, we conducted a prospective study of 26 patients to validate the use of the cutoff ADC value obtained in the study's first stage. The sensitivity, specificity, and overall accuracy for differentiating malignant from benign endometrial lesions based on the cutoff ADC value obtained earlier were as follows: radiologist 1 attained 86.67%, 100.0%, and 92.31%, respectively; radiologist 2 attained 86.67%, 91.0%, and 88.5%, respectively. Our results suggest that ADC values could be a potential biomarker for use as a quantitative and qualitative tool for differentiating between early-stage endometrial carcinomas and benign mimickers.

## INTRODUCTION

Endometrial cytology, biopsy, and curettage have been the mainstays for accurately diagnosing lesions in the endometrial cavity. As these procedures are commonly performed in a blind manner, however, they do not always provide a definitive diagnosis. In addition, they are difficult to perform in patients with vaginal or cervical stenosis [1–3]. Transvaginal sonography has been proposed as the first-line diagnostic tool for evaluating endometrial thickness, but operator experience, the position of the uterus, and vaginal anomalies limit its usefulness. Because of its excellent soft tissue contrast resolution, multi-planar imaging capability, and post-processing tools, magnetic resonance imaging (MRI) is increasingly being applied for diagnosing endometrial diseases and as a problem-solving tool when there is a diagnostic dilemma [4–6].

Clinically, however, using conventional MRI to differentiate endometrial carcinoma from benign mimickers, such as polyps and submucosal myomas, remains challenging [7]. In contrast, diffusion-weighted imaging (DWI) is based on diffused, random motion of water molecules and provides information about cell density of a given tissue and the integrity of cell membranes [8]. It can be quantified using an apparent diffusion coefficient (ADC) or qualitatively assessed in biological tissues [9, 10].

ADC values have been shown to be decreased in various tumors [11–16]. The assessment of endometrial carcinomas versus benign lesions in the endometrial cavity using DWI and 1.5T MRI has been reported [14, 17–19]. As 3T MRI scanners are increasingly used in clinical practice, it is necessary to investigate the cutoff ADC value for differentiating endometrial carcinomas from benign mimickers at 3T MRI. To our knowledge, there are few data prospectively validating the diagnostic performance of the cutoff ADC value for lesions in the endometrial cavity using 3T MRI.

In this study, we first obtained an ADC cutoff value via a retrospective study at 3T MRI. The ADC value was then validated prospectively to differentiate stage I endometrial carcinoma and its benign mimickers. This study aimed to investigate the feasibility of using the ADC value to differentiate early-stage endometrial carcinomas from benign mimickers.

## RESULTS

**Figure 1:**
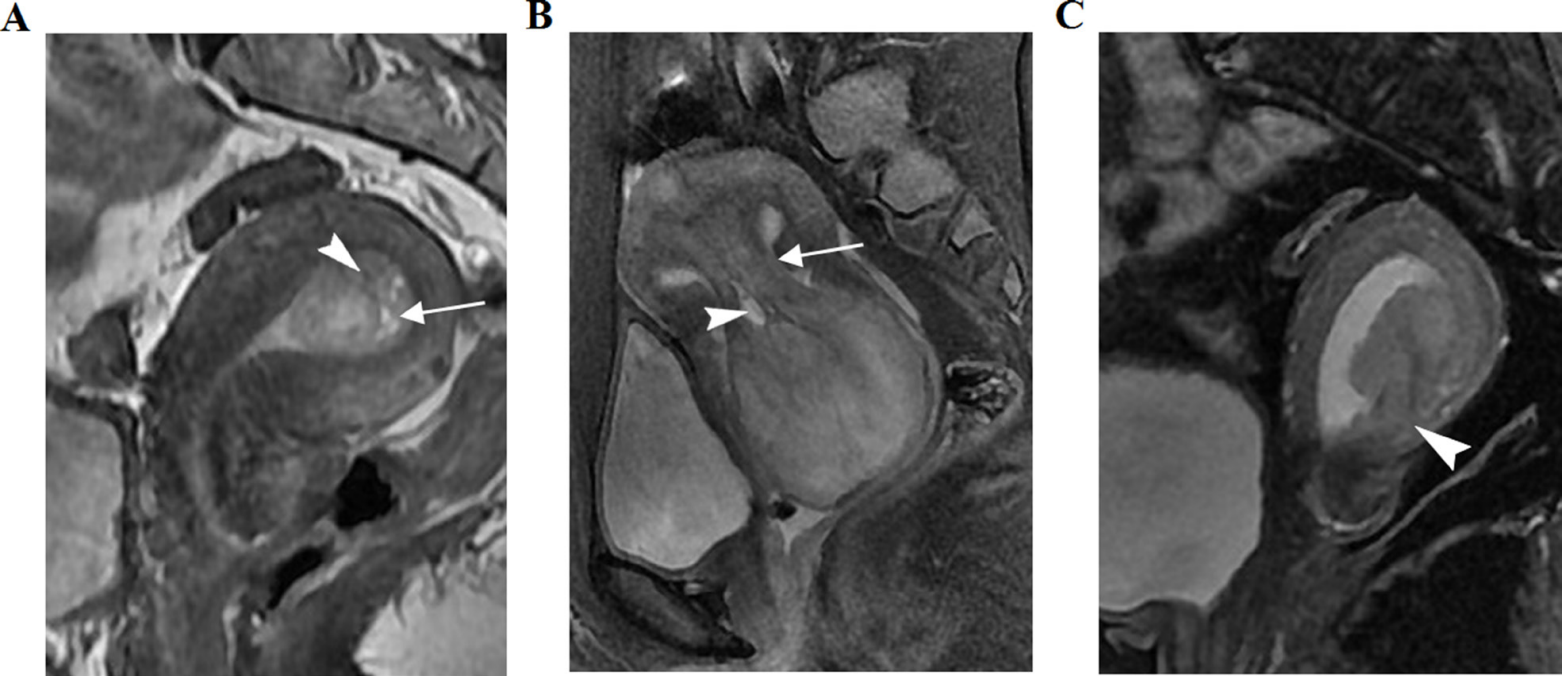
Three women with histopathologically proven uterine polyps, submucosal myoma, and stage I endometrial carcinoma, respectively, on sagittal T2-weighted imaging (T2WI) (**A**) Polyp shows slightly heterogeneous hyperintensity, with fibrous cores (arrow) and small, high-intensity intratumoral cysts (arrowhead). (**B**) Submucosal myoma shows slight hyperintensity compared with the normal outer myometrium. A peduncle (arrow) from the uterine fundus and intratumoral cysts (arrowhead) are seen. (**C**) Intensity of a stage Ib endometrial carcinoma was equal to that of the normal outer myometrium. Local myometrium was not consistent (arrowhead).

**Figure 2:**
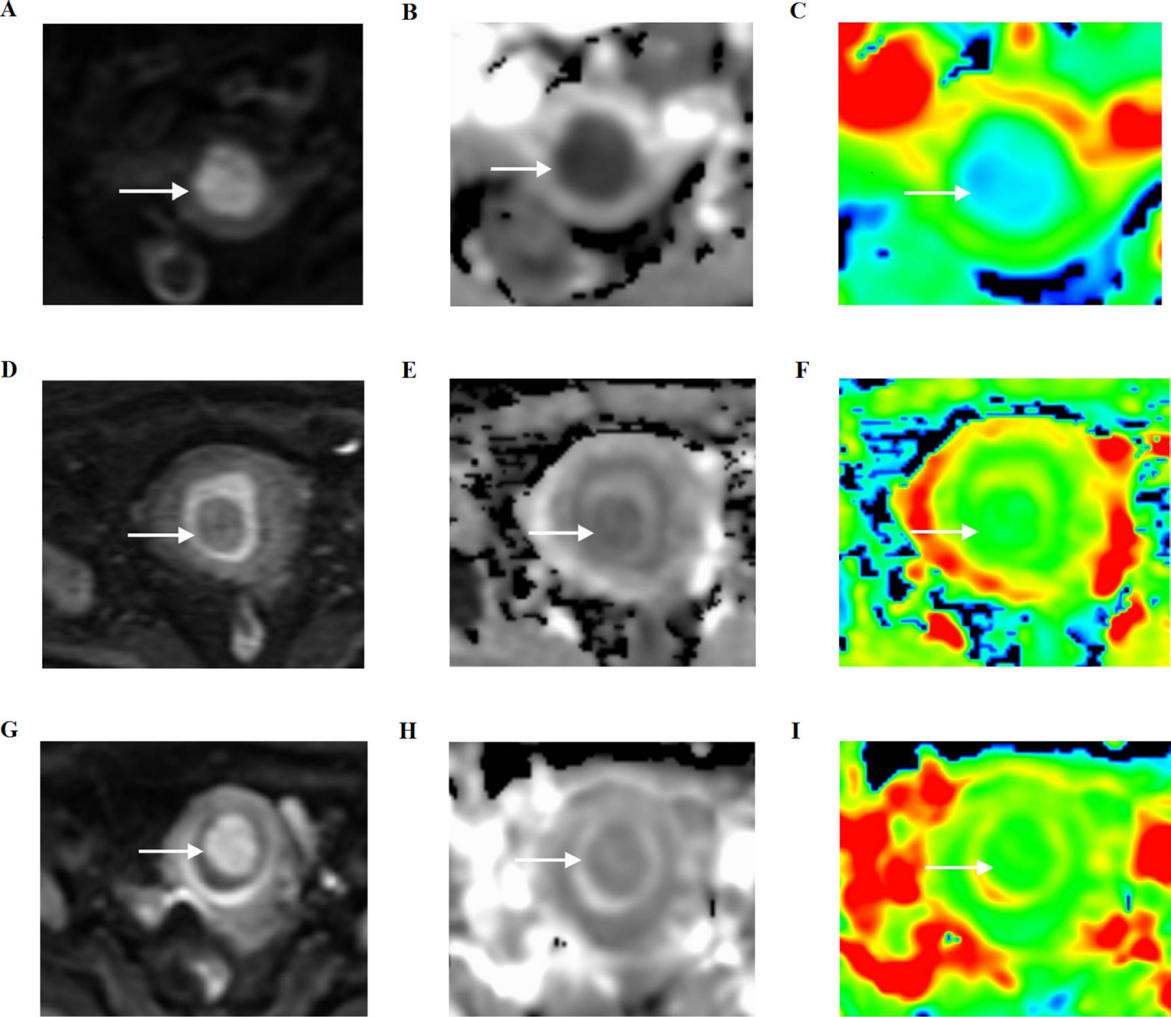
Three women with histopathologically proven stage I endometrial carcinoma, endometrial polyp, and a submucosal myoma, respectively, on axial diffusion-weighted imaging (DWI) and apparent diffusion coefficient (ADC) maps (**A**–**C**) Stage I endometrial carcinoma shows hyperintensity on DWI (arrow), hypointensity, and blue foci on the ADC map (arrow). (**D**–**F**) Endometrial polyp shows hypointensity on DWI (arrow), light hypointensity, and blue foci on the ADC map (arrow). (**G**–**I**) Submucosal myoma shows light hyperintensity on DWI (arrow), light hypointensity, and green foci on the ADC map (arrow).

**Figure 3:**
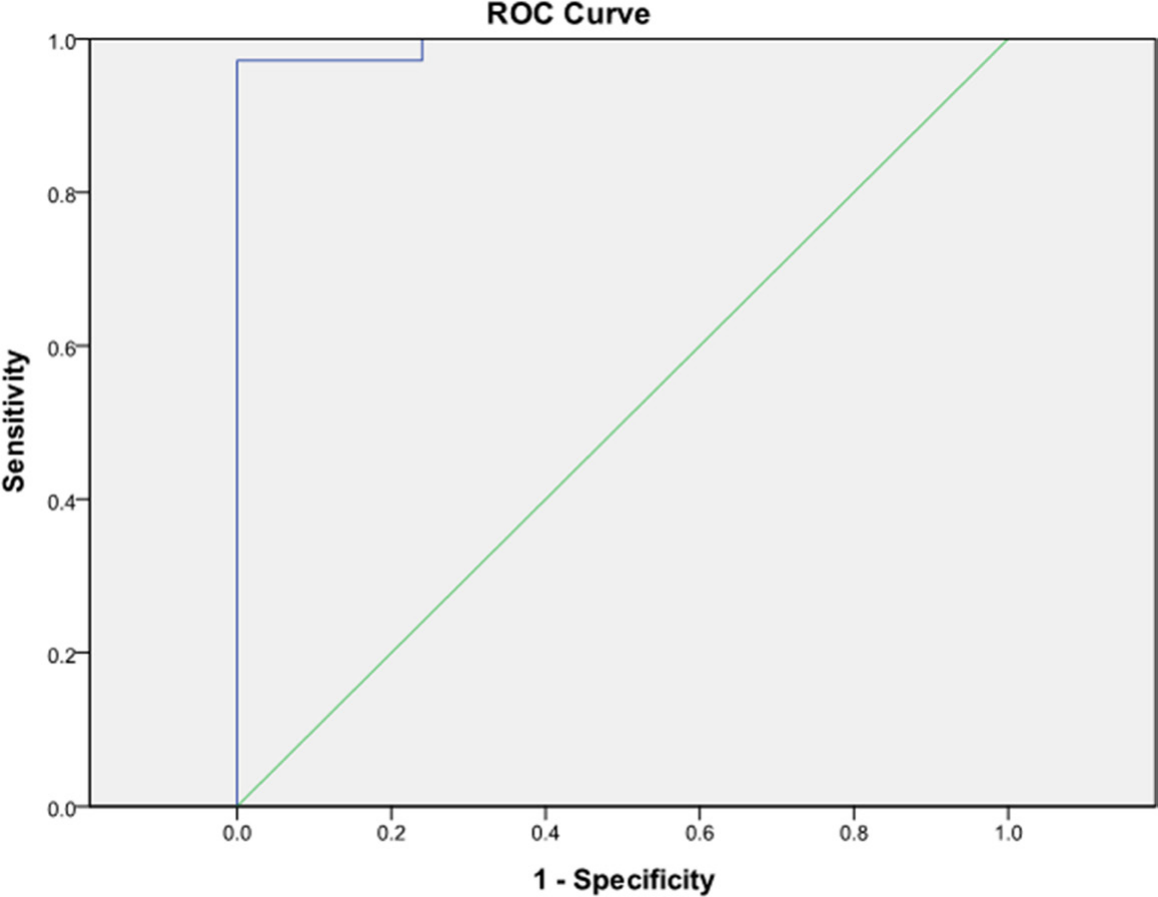
Receiver operating characteristic curve analysis showed an area under the curve for ADC values of 0.993 (95% confidence interval 0–1) Cutoff value differentiating stage I endometrial carcinoma from benign lesions was 0.98 × 10^−3^ mm^2^/s.

For the first stage of the study (May 2012 to September 2014), we enrolled 60 patients (ages 45–75 years, mean 58 years) with lesions in the uterine endometrial cavity. The histological types and frequencies of the lesions were as follows: stage Ia endometrial carcinomas (*n* = 22); stage Ib endometrial carcinomas (*n* = 3); endometrial polyps (*n* = 15); submucosal leiomyomas (*n* = 20). These lesions appeared as isointense to slightly high signal intensity compared with the normal myometrium on T2-weighted imaging (T2WI). In all, 35 benign endometrial lesions presented with slightly low or intermediate signal intensity on T2WI. Among 25 endometrial cancers, 11 had intratumoral cystic changes and 3 had fibrous cores, whereas among the 35 benign lesions, only 3 showed intratumoral cystic changes and 3 had fibrous cores. T2WI revealed four pedunculated submucosal leiomyomas. Thus, the presence of these three features (signal intensity, cystic changes, fibrous cores) was not significantly different between endometrial carcinomas and benign uterine lesions (*P* = 0.079). When combining the results of T2WI and DWI, however, myometrial invasion was seen in 22 of the 25 endometrial carcinomas. No myometrial invasion was found in the benign lesions (Figure 1). All 25 stage I endometrial carcinomas showed high signal intensity on DWI as well as hypointensity (25/25) and blue foci (25/25) on the ADC map. All 35 benign endometrial lesions showed slight hypointensity or hyperintensity on DWI as well as hypointensity (35/35) and green foci (30/35) on the ADC map. Some blue foci were seen in one polyp and four submucosal myomas (Figure 2). The ADC value (×10^−3^ mm^2^/s) of stage I endometrial carcinomas (0.80 ± 0.10) was significantly lower than that of the benign endometrial lesions (1.43 ± 0.25) (*P* = 0). The mean ADC value for the endometrial polyps (1.53 ± 0.23) was significantly higher than that for the submucosal leiomyomas (1.35 ± 0.25) (*P* = 0.032). The area under the receiver operating characteristic (ROC) curve was 0.993. The cutoff ADC value obtained was 0.98 ×10^−3^ mm^2^/s for achieving the highest accuracy in differentiating stage I endometrial carcinomas from benign endometrial lesions (Figure 3). The sensitivity, specificity, and overall accuracy for diagnosing of stage I endometrial carcinomas were 100% (25/25), 97.1% (34/35), and 98.3% (59/60), respectively, whereas the accuracy range based on the various cutoff ADC values in previous studies was 83.3%–93.3% (Table 1).

**Table 1:** ADC values from the literature and this study

Authors	Magnetic field strength	b-values (s/mm^2^) applied	Suggested cutoff ADC value (×10^−3^ mm^2^/s)	Sensitivity (%)	Specificity (%)	Accuracy (%)
Kilickesmez et al. [15]	1.5T	0, 500, 1000	1.05	100	88.6	93.3
Fujii et al. [14]	1.5T	0, 1000	1.15	100	85.7	91.7
Takeuchi et al. [17]	1.5T and 3T	0, 800	1.2	100	82.9	90.0
Karakas et al. [20]	1.5T	0, 500, 1000	0.908	88	97.1	93.3
Bharwani et al. [19]	1.5T	0, 50, 100, 250, 500, 750	1.28	100	71.4	83.3
This study	3T	0,1000	0.98	100	97.1	98.3

ADC: apparent diffusion coefficient.

For the second stage of the study (October 2014 to November 2015), the study group included 26 patients (ages 30–70 years, mean 60 years) with lesions in the uterine endometrial cavity. The histological types and frequencies of the lesions were as follows: stage Ia endometrial carcinoma (*n* = 9); stage Ib endometrial carcinoma (*n* = 1); stage IIa endometrial carcinomas (*n* = 5); endometrial polyps (*n* = 4); submucosal leiomyomas (*n* = 7). The kappa value for agreement of the measured ADC values between two radiologists was 0.846 (> 0.75). The sensitivity, specificity, and overall accuracy of radiologist 1 (based on the cutoff ADC value obtained in stage 1 of the study) were 86.67% (13/15), 100.0% (11/11), and 92.31% (24/26), respectively, and those of radiologist 2 were 86.67% (13/15), 91.0% (10/11), and 88.5% (23/26), respectively.

## DISCUSSION

Previous researchers [14, 15, 17, 19, 20] tried to find a simple, accurate way to differentiate between benign and malignant endometrial lesions. They used various cutoff ADC values as imaging biomarkers to achieve this goal. To our knowledge, however, few studies used 3T MRI, and few validated the effectiveness of these cutoff ADC values. The present study was performed in two stages to, first, obtain a cutoff ADC value at 3T MRI and, second, prospectively validate use of the ADC value.

During the first stage, we obtained a theoretical cutoff ADC value of 0.98×10^−3^ mm^2^/s for differentiation between benign and malignant endometrial lesions at 3T MRI. Using the cutoff ADC value during the second stage of the study, we prospectively assessed its sensitivity, specificity, and accuracy for distinguishing malignant endometrial lesions from benign lesions. We found that the sensitivity, specificity, and accuracy for diagnosing endometrial carcinomas were high, indicating that the cutoff ADC value could be used as an imaging biomarker to differentiate between benign and malignant endometrial lesions.

Conventional MRI sequences, such as T1WI and T2WI, were generally use for evaluating endometrial lesions. Park et al. [7] reported that, on T2WI, intratumoral cystic changes and fibrous cores were seen less frequently in stage I endometrial carcinomas than in benign endometrial masses. In contrast, our study showed that fibrous cores and intratumoral cystic changes were seen more frequently in endometrial polyps than in endometrial cancer, in accord with Grasel et al.'s study [21]. Pedunculated lesions are usually considered benign. In the present study, only four benign lesions of the endometrium (4/35) were characterized as pedunculated. Hence, morphological features on T2WI might not be helpful in differentiating these lesions.

DWI is a functional imaging method based on the quantification of increased or restricted microscopic diffusion motions of tissue water molecules [14]. Increased tumor cellularity, which can restrict water diffusion, therefore decreases ADC values. Malignant lesions frequently have greater cellularity than benign lesions. Thus, ADC values can contribute to distinguishing between benign and malignant lesions [22–27]. Various cutoff ADC values have been used to differentiate malignant from benign uterine lesions [14, 15, 17, 19, 20]. These cutoff ADC values ranged from 0.908 × 10^−3^ mm^2^/s to 1.28 × 10^−3^ mm^2^/s. In this study, by applying the cutoff ADC value of 0.98 × 10^−3^ mm^2^/s, we achieved the greatest accuracy (98.3%).

There are some major differences between previous studies and the present one. First, the previously published cutoff values were obtained at 1.5T MRI in four studies [14, 15, 19, 20], and Takeuchi et al. [17] used both 1.5T and 3T systems. In this study, we performed 3.0T MRI in all patients to enhance the signal-to-noise ratio. A phantom study [28] confirmed that ADC values vary at 1.5T and 3T. Second, using different b values could affect the calculation of ADC values, causing variable results. Thus, it is difficult to establish a universal threshold for the ADC value. Third, because benign endometrial lesions and stage I endometrial carcinomas are similar, accurate diagnosis is difficult. Hence, all endometrial malignant lesions were known to be stage I endometrial carcinomas during the first stage of of this study. In contrast, previous studies included all endometrial carcinoma stages or carcinosarcomas. Lastly, the ADC value may relate to individual changes, such as age and body temperature, causing interpatient variation [29, 30].

In this study, we found that the ADC value for endometrial polyps was significantly higher than that for submucosal leiomyomas. Leiomyomas are composed of smooth muscle cells and collagen, which causes lower ADC values [31]. They are also associated with degeneration (e.g., hyalinization, edema), which occurs in more than 50% of leiomyomas [32]. Hyalinization leads to accumulation of homogeneous eosinophilic bands in the extracellular space, narrowing this space and thus decreasing ADC values [33]. In contrast, edema leads to increased ADC values because of widening of the extracellular space. The ADC value for leiomyoma is related to these histological findings and is thus complicated. Endometrial polyps contain endometrial glands and stroma or smooth muscle tissue. Cystic glandular hyperplasia generally develops in polyps. These changes can decrease cellularity and increase movement of water molecules, thus promoting increased ADC values [14, 34]. Further studies in larger samples are needed to investigate a cutoff ADC value for the differential diagnosis of endometrial polyps and submucosal leiomyomas.

The cutoff ADC value obtained during the first stage of the study was prospectively applied in the second stage to validate its effectiveness. The high kappa value for agreement regarding measured ADC values between two radiologists indicates that this ADC value could be used reliably to assess endometrial lesions. The sensitivity, specificity, and accuracy of differentiating malignant endometrial lesions from benign lesions were high. These results indicated that the ADC value acquired using DWI could be a potential quantitative and qualitative biomarker to differentiate benign and malignant endometrial lesions. ADC values, however, might not be measured accurately in some small endometrial masses. In this situation, DWI could enhance its diagnostic capability if it is combined with dynamic contrast enhancement.

Our study has some limitations. First, endometrial carcinomas, polyps, and submucosal myomas < 10 mm or thick endometrium were not included in this study to ensure relatively reliable results. Second, the sample size for the second (prospective) stage of the study was relatively small to validate the cutoff ADC value obtained from the first (retrospective) stage of the study. Nevertheless, a PubMed search revealed no other publications that prospectively validated the use of ADC as a biomarker in the endometrial cavity.

The ADC values for stage I endometrial carcinomas were significantly lower than those for benign lesions, or “mimickers.” The ADC value obtained with DWI could be a potential biomarker when used as a quantitative and qualitative tool for differentiating between early-stage endometrial carcinomas and their benign mimickers.

## MATERIALS AND METHODS

The study was designed into two stages. The first stage, a retrospective study, was performed to evaluate the diagnostic value of ADC and obtained a theoretical ADC cutoff value for differentiating benign and malignant endometrial lesions. The second stage, a prospective study, was performed to validate the ADC cutoff value obtained for differentiating between benign and malignant endometrial lesions.

### Subjects

Our institutional review board granted a waiver for consent because of the anonymous nature of this study. A total of 131 consecutive patients were identified. During the first stage, we excluded 6 patients with stage II–IV endometrial carcinomas and 5 patients whose imaging was of such poor quality we were unable to make a diagnosis. We excluded 10 patients due to endometrial hyperplasia with no mass in the endometrial cavity and 5 patients whose endometrial lesions were < 1.0 cm diameter (2 endometrial hyperplasia, 3 stage Ia endometrial carcinoma). During the second (prospective) stage of the study, we excluded 4 patients because their clinically suspicious endometrial masses were simply thick endometrium as shown on MRI, 5 patients whose endometrial lesions were < 1.0 cm diameter, and 10 patients whose lesions were not histologically confirmed. Finally, 86 patients (ages 30–75 years, mean 60 years) with International Federation of Gynecology and Obstetrics (FIGO) stage I endometrial carcinomas, submucosal leiomyomas, and polyps were enrolled in this study. All tumors were later confirmed histopathologically. Hysteroscopic polypectomy or myomectomy was performed for benign lesions and hysterectomy for malignant lesions. All patients underwent conventional MRI and DWI with b values of 0 s/mm^2^ and 1000 s/mm^2^, respectively, before surgery. These lesions were assessed by two blinded experienced radiologists in gynecological imaging.

### Imaging protocol

MRI was performed by a 3T scanner (HDXT; General Electric Medical Systems, Milwaukee, WI, USA) with an 8-channel torso phased-array coil. All patients were examined with T1-weighted imaging (T1WI), T2-weighted imaging (T2WI), and DWI. Parameters were as follows: (1) T1WI in axial oblique (to the corpus) planes: repetition time (TR)/time to echo (TE) 500–600/7–10 ms, field of view (FOV) 380 × 380 mm, section thickness 5.0 mm, intersection gap 3 mm; (2) short time inversion recovery T2WI in axial, sagittal, and coronal planes: TR/TE 2900–3000/70–80 ms, FOV 280–380 × 380 mm, section thickness 5 mm, intersection gap 3.0–5.0 mm. (3) Axial DWI of the pelvis: TR/TE 5000/66 ms, FOV 380 × 380 mm, matrix 96×130, section thickness 5 mm, b = 0 and 1000 s/mm^2^, respectively.

### Imaging analysis

MRI images were retrospectively and blindly evaluated by two radiologists during the first stage of the study, with discrepancies resolved by a consensus discussion. On DWI, high-, iso-, or low-signal lesions, compared with the normal endometrium signals, were identified. Myometrial invasion was diagnosed when the endometrial-myometrial junction was disrupted. We obtained a cutoff ADC value for differentiating malignant and benign lesions from ROC curve. Other cutoff ADC values were obtained from published studies [14, 15, 17, 19, 20], which along with our ADC, were used to calculate corresponding the sensitivity, specificity, and accuracy. During the second stage of the study, the cutoff ADC value obtained during the first stage was prospectively applied to the lesions to predict the diagnosis of either endometrial malignancy or a benign lesion.

### Imaging post-processing

**Figure 4:**
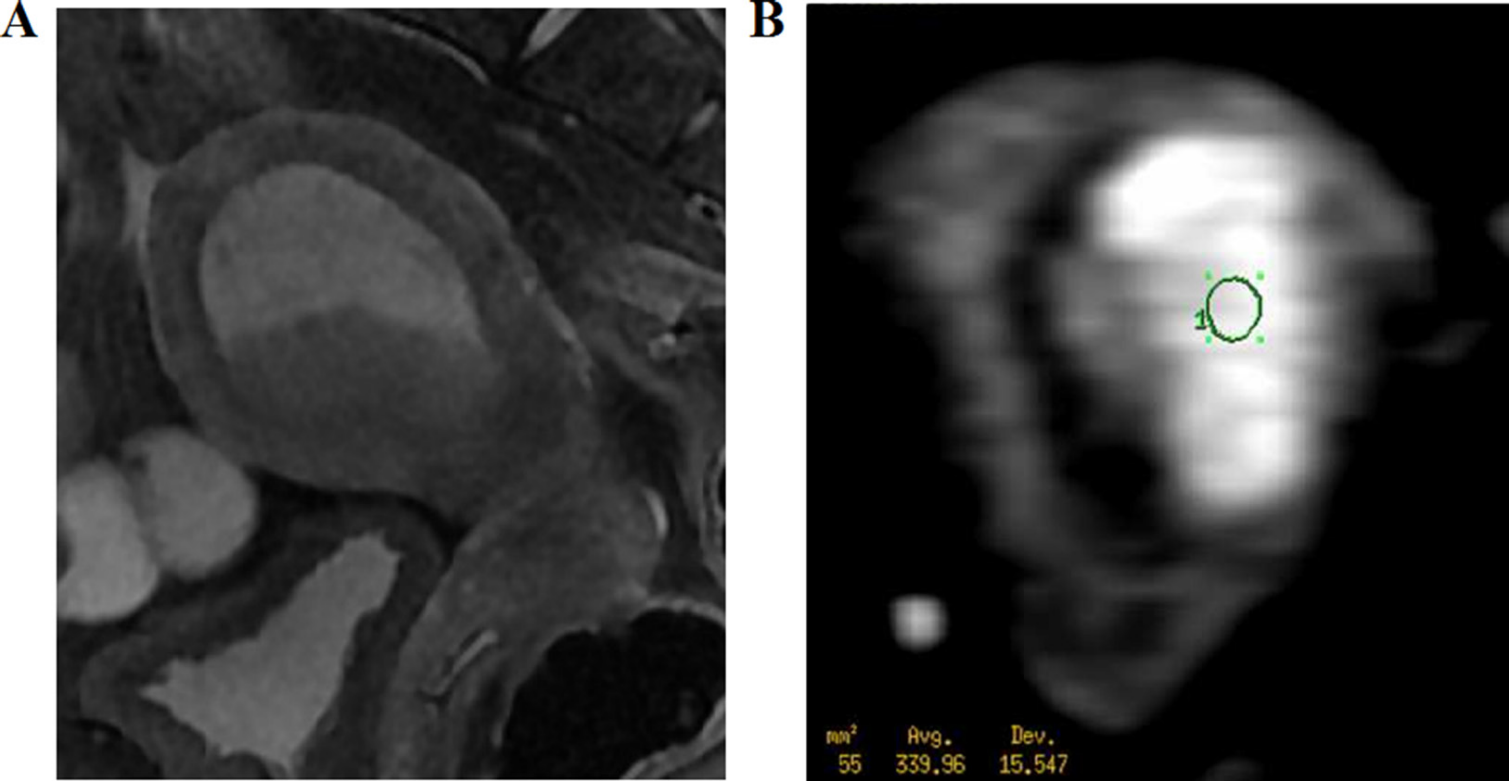
A 62-year-old woman with stage I endometrial carcinoma (**A**) Sagittal T2WI shows the carcinoma with slightly high intensity (arrow). (**B**) Axial DWI shows a carcinoma with hyperintensity. The region of interest (55 mm^2^) was placed at the center of the lesion.

ADC maps were generated from a built-in software package (GE Func Tool Version 4.5) using b = 0 and b = 1000 s/mm^2^ images. For each patient, a circular region of interest (ROI) (55 mm^2^) was created on central slices that contained the largest portion of the tumor. Each ROI was positioned on the solid area of the lesion while excluding any cystic and/or necrotic area and any endometrial tissue (Figure 4). The ADC values were then calculated automatically. The ROI was measured three times and the mean value recorded.

### Statistical analysis

The Kolmogorov–Smirnov test was used to determine the normality of the data distributions of the ADC values for both stages of the study. For the first stage, Student's *t*-test was applied to compare the ADC values of malignant and benign tumors and between endometrial polyps and submucosal leiomyomas. The ROC curve was used to obtain a cutoff ADC value for differentiating stage I endometrial carcinomas from benign lesions. The sensitivity, specificity, and accuracy were also calculated. For the second stage of the study, the cutoff ADC value attained during the first stage was prospectively applied to lesions to calculate its sensitivity, specificity, and accuracy to validate its diagnostic performance. The kappa test was used to assess the agreement between the two radiologists. SPSS software 18.0 (SPSS, Chicago, IL, USA) was used for the statistical analyses. *P* < 0.05 was considered to indicate statistical significance.

## Abbreviations

ADC: apparent diffusion coefficient
AUC: area under the curve
MRI: magnetic resonance imaging
DWI: diffusion-weighted imaging
TR/TE: repetition time/time to echo
FOV: field of view
ROC: receiver operating characteristic
ROI: region of interest.
